# Regulatory Role of Apoptotic and Inflammasome Related Proteins and Their Possible Functional Aspect in Thiram Associated Tibial Dyschondroplasia of Poultry

**DOI:** 10.3390/ani12162028

**Published:** 2022-08-10

**Authors:** Muhammad Fakhar-e-Alam Kulyar, Wangyuan Yao, Quan Mo, Yanmei Ding, Yan Zhang, Jindong Gao, Kewei Li, Huachun Pan, Shah Nawaz, Muhammad Shahzad, Khalid Mehmood, Mudassar Iqbal, Muhammad Akhtar, Zeeshan Ahmad Bhutta, Muhammad Waqas, Jiakui Li, Desheng Qi

**Affiliations:** 1 College of Veterinary Medicine, Huazhong Agricultural University, Wuhan 430070, China; 2 Department of Animal Nutrition and Feed Science, College of Animal Science and Technology, Huazhong Agricultural University, Wuhan 430070, China; 3 Faculty of Veterinary and Animal Sciences, The Islamia University of Bahawalpur, Bahawalpur 63100, Pakistan; 4 College of Veterinary Medicine, Chungbuk National University, Cheongju 28644, Korea; 5 Faculty of Veterinary & Animal Sciences, University of Poonch Rawalakot, Rawalakot 12350, Pakistan; 6 College of Animals Husbandry and Veterinary Medicine, Tibet Agricultural and Animal Husbandry University, Linzhi 860000, China

**Keywords:** poultry, tibial dyschondroplasia, thiram, apoptosis, inflammasome, chondrocytes

## Abstract

**Simple Summary:**

Tibial dyschondroplasia (TD) is a metabolic disorder that impairs bony and cartilage processes. It is common in broilers due to the consumption of thiram, especially in the industrial and agriculture zones. During the condition, cartilage does not only seem to develop ossification during its occurrence but also causes lameness, mortality, and moral convictions in commercial poultry. Moreover, it has been characterized as an economically significant condition since it causes carcass damage due to the involvement of different biological pathways that lead to a particular change in the chondrocytes. These entire cellular pathways are interconnected through various cellular inputs, including anti-apoptotic, pro-apoptotic, and executioner caspases that modulate the other essential chondrogenic proteins (collagen and aggrecan), extracellular metalloproteinases, and NLRP3 base inflammasome.

**Abstract:**

Tibial dyschondroplasia debilities apoptotic and inflammasomal conditions that can further destroy chondrocytes. Inflammasomes are specialized protein complexes that process pro-inflammatory cytokines, e.g., interleukin-1β (IL-1β) and IL-18. Moreover, there is mounting evidence that many of the signaling molecules that govern programmed cell death also affect inflammasome activation in a cell-intrinsic way. During the last decade, apoptotic functions have been described for signaling molecules involving inflammatory responses and cell death pathways. Considering these exceptional developments in the knowledge of processes, this review gives a glimpse of the significance of these two pathways and their connected proteins in tibial dyschondroplasia. The current review deeply elaborates on the elevated level of signaling mediators of mitochondrial-mediated apoptosis and the inflammasome. Although investigating these pathways’ mechanisms has made significant progress, this review identifies areas where more study is especially required. It might lead to developing innovative therapeutics for tibial dyschondroplasia and other associated bone disorders, e.g., osteoporosis and osteoarthritis, where apoptosis and inflammasome are the significant pathways.

## 1. Introduction

Cellular and molecular pathways regulate bone formation and growth. Any variation from the normal process may result in bone abnormalities, which pose a significant economic challenge to the poultry business [1]. The control of bone formation and growth seems complicated, with several layers of interaction between the regulatory factors [2]. Chondrocytes’ formation and differentiation occur on the growth plate in a particular region. Pre-hypertrophic chondrocytes form a columnar layer once proliferating chondrocytes stop replicating. These phases are determined by the cellular phenotype and extracellular matrix metalloproteinase (ECM) proteins. Columnar cells, pre-hypertrophic cells, and hypertrophic cells express distinct transcription factors and ECM proteins as they progress through the embryonic stages [3]. As a result, interactions between these essential processes in the growth plate become necessary for appropriate long bone development [4].

Tibial dyschondroplasia is among the remarkably prevailing skeletal abnormalities affecting young poultry birds [5]. The prevalence of this tibiotarsal bone condition has increased by 30% in the flock at broiler farms. Due to the majority of its symptoms being sub-clinical [6], it is often difficult to adequately detect the prevalence of TD at these farms; as a result, farmers frequently find it easy to let their guard down. In fact, broilers with TD experience leg weakness, limited motion, and even walking difficulties. Broilers are more likely to sustain fractures during the feeding process, which negatively impacts the welfare of the birds and reduces production, which further causes significant financial loss for the poultry industry. Various researchers worldwide have constantly focused on the etiology and prevention of TD [7,8]. In most cases, nutritional, ecological, and genetic factors have been implicated in its etiology [9]. For instance, soybean meal in feeding has been associated with TD pervasiveness, along with further concerns, including ergocalciferol insufficiency, hyperthyroidism (overactive thyroid), and abnormal levels of biological parameters such as interleukin-1β and nitric oxide [10]. Moreover, according to some studies, copper deficiency, fusarochromanone, excessive dietary levels of cysteine and homocysteine, metabolic acidosis [11], vitamin D deficiency [12], disbalance of calcium and phosphorus [13], and thiram contamination [14] may also cause the condition. The condition of TD has been linked to aberrant ossification and prolongation of tibial growth plates (GP) as a result of reduced chondrocyte propagation and differentiation [15]. An ideal cartilage matrix has enough blood supply and mineralization; however, this is not always the case for TD [16]. During TD conditions, chondrocytes are premature and more prominent than usual because of pre-hypertrophic enlargement with avascular ossein zones in cartilage [17].

Pesticides are widely used in agriculture to eradicate or control many agricultural bugs, herbicides, and diseases that may harm crops and animals. On the other side, pesticides have become a hazard due to their toxicity. Living organisms may be exposed precisely or periphrastically over the food chain, air, soil, and water [18]. Thiram (Tetramethyl thiuram disulfide) is a dithiocarbamate pesticide and fungicide commonly used in horticulture to treat grains for seed protection and preservation [19]. It has a lipophilic character that can effortlessly combine with cell membranes to induce cytotoxicity, bone formation problems, cartilage damage, and immunological downturns. It may also cause membrane disruption, bone biosynthetic pathway inactivation, and angiogenesis inhibition [20]. So, it is highly associated with the induction of TD, with symptoms that resemble commonly occurring tibial dyschondroplasia. Additionally, earlier research has shown that TH (thiram) may cause TD in chickens at the dose rate of 50 mg/kg [5,21]. Moreover, it has been frequently mobilized to imitate TD in numerous research trials [22,23,24,25]. Our prior studies indicate that thiram induces apoptosis in chondrocytes raising the number of apoptotic chondrocytes inside the osteogenesis area [26,27,28]. Besides, thiram inhibits angiogenesis within the GP, reducing chondrocytes function and osteogenesis [29]. Among thiram’s most visible and damaging effects is a bone cartilage disorder in broilers when fed thiram-containing diets [24,29]. During this disorder, non-mineralized avascular cartilage accumulates in the growth plates of the proximal tibia, resulting in lameness [30].

## 2. Prospective Tibial Cartilage Development

**Figure 1:**
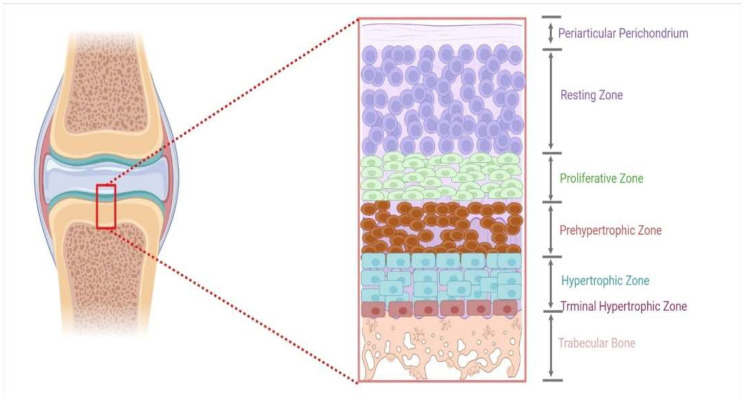
An illustration of a growth plate’s different zones.

The growth and development of long bones in poultry are accomplished by chondrocytes and a matrix composed of highly organized growth plates [31]. These cells in the growth plate can be subdivided into five phases for endochondral ossification (EO) [32]. OE is a term used to describe the process of bone formation. It is a process that includes the continuous replacement of growing cartilage to generate a thick bony structure [33]. The chondrocytes multiply, grow, and die during the ossification process, while the extracellular matrix that builds during this process is subsequently invaded by the vascular system and various bone cells, forming bone over cartilage matrix remnants [34]. The process occurs in three unique locations: the physis, the epiphysis, and the cuboidal. During bone development, chondrocytes may be subdivided into layers or zones in which the hypertrophic zone is the most important for the formation of endochondral ossification [35,36]. Chondrocytes are formed in this area by final differentiation of the proliferating zone farthest from the epiphyseal plate. When these cells stop proliferating, they enlarge, profoundly impacting the development process [37,38]. Once the bone has developed to both ends, the hypertrophic zone forms at the bottom of the growth plate, between the preceding propagating cell layers and the epiphyseal bone (Figure 1) [35]. Here, the chondrocytes are surrounded by an extracellular matrix, which eventually mineralizes in the zone of preliminary calcification. After the invasion of the chondrocyte columns by the metaphyseal vascular system, bone grows on the remaining columns of hardened cartilage. The combination of hardened cartilage and undeveloped bone is then progressively reshaped to become mature bone [39].

Each growth plate is a multilayer sandwich structure organized into four distinct zones: reserve, proliferating, transformational, and degeneration zones [40]. Immature cells in the growth plate are included in the resting zone because they are located near the epiphysis. This zone comprises tiny, homogeneous, compactly placed chondrocytes that appear alone or in couples and are positioned inside the reserve zone (also called resting or germinal area). This zone is further distinguished by a low proliferation rate, proteoglycan, and collagen type II production [41,42]. The proliferating zone is the next layer down from the reserve area. Chondrocytes are flattened and well-separated into columns in this area. During mitosis, cells only divide at the bottom of a column. Collagen production in the true germinal layer has risen significantly in type II and type XI [43]. The transformation zone, which lies beneath the proliferative zone, is divided into a top and bottom hypertrophic zone and a degenerative zone. Chondrocytes at this stage are distinguished by their lack of cellular proliferation and decreased DNA synthesis [15,43].

## 3. Growth Plate Associated Tibial Dyschondroplasia in Poultry

**Figure 2:**
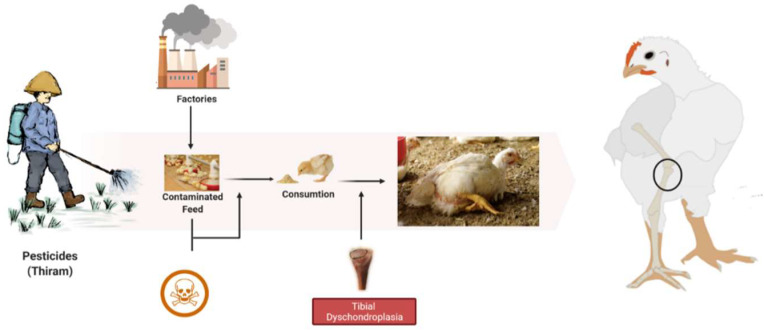
The root cause of tibial dyschondroplasia in broilers.

During TD condition, the chondrocytes in the growth plate region are unorganized, having fewer blood vessels with lesions in proliferative and hypertrophic zones [29]. These lesions include avascular, noncalcified tissue and soft cartilage. Histologically, hypertrophic zone enlarges and combines with avascular cartilage zones [17]. Thiram is highly associated with the induction of TD, with symptoms resembling tibial dyschondroplasia (Figure 2). Additionally, earlier research has shown that TH (Thiram) may cause TD in chickens. Moreover, it has been frequently mobilized to imitate TD in numerous research trials [14,22,44]. Our prior studies indicate that thiram induces apoptosis in chondrocytes, raising the number of apoptotic chondrocytes inside the osteogenesis area [27,45]. Besides, thiram inhibits angiogenesis within the GP, reducing chondrocytes function and osteogenesis [23,29].

## 4. Role of Different Proteins in the Pathogenesis of TD

Research on disease pathogenesis and treatment involves comparing a diseased condition to a healthy one. The discovery of differentially regulated proteins in a disease seems to be a particular focus of inquiry. Those proteins might be a promising biomarker in pharmacological and clinical research. This kind of data might be helpful after combining with other biological data to establish a disease’s target picture. Too far, a plethora of research has demonstrated the essential proteins encode in combat against TD. Following are some significant proteins involved in tibial dyschondroplasia.

### 4.1. Role of Chondrogenic Marker Proteins

The damaged articular cartilage has a deficient ability to mend itself from causing any impairment [46]. Collagen II and aggrecan are two of the bones’ most crucial extracellular matrix components [47]. Any defect in collagen (type II) results either in approximate or quantitative alteration, depending on the mutant site. As a result, the clinical symptoms of collagen II abnormalities range from neonatal mortality to minor skeletal dysplasia [48]. Similarly, aggrecan is also a significant proteoglycan in the articular cartilage, expressed by chondrocytes. This protein is essential in chondro-skeletal morphogenesis during development [49]. Furthermore, due to its glycosaminoglycan concentration, aggrecan plays an integral part in producing the cartilage’s persistent negative charge, resulting in its water-attracting qualities [50].

**Figure 3:**
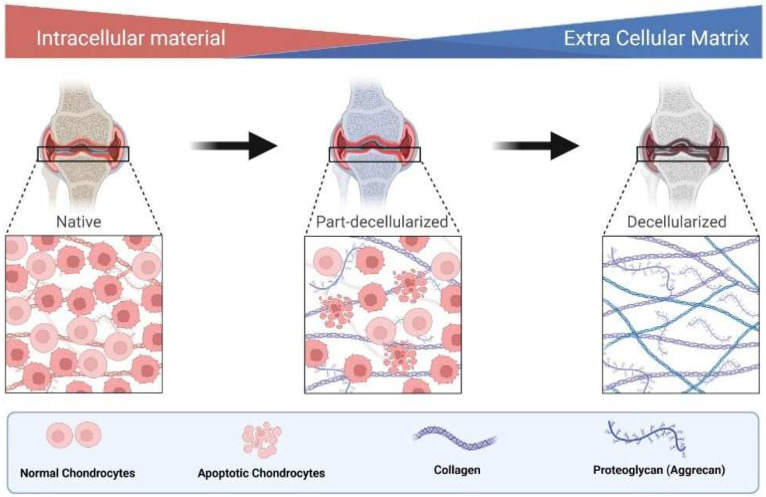
Role of collagen and aggrecan mediated ECM in decellularization.

In this way, these macromolecules are helpful in the process of “decellularized extracellular matrix” (dECM), which is directly connected with the maintenance of the native environment for promoting cell proliferation and differentiation [51] (Figure 3). Hence, reducing these macromolecules may affect normal and pathological bone development [52].

### 4.2. Role of CD147 (EMMPRIN/Basigin) Protein

The Cluster of differentiation 147 (CD147) protein decrypted by the BSG gene is an immunoglobulin superfamily member interacting with the cell membrane. It has a type I integral membrane binding site with 269 amino acids that features two ig domains at the N-terminal [53]. It is also known as an extracellular matrix metalloproteinase inducer (EMMPRIN) or basigin [54], located on the exterior of apoptotic cells. It may stimulate the transcription or activation of matrix metalloproteinase (MMP) in neighboring mesenchymal and malignant cells, hence promoting tumor invasion [55]. Recent investigations have shown that CD147 is expressed in malignant cells and various other cells, including fibroblast and keratinocytes [56]. Additionally, it is vital to replenish the extracellular matrix components required for continuous bone formation [57]. Furthermore, it is involved in cellular reflexes, metabolism, inflammation, distant metastasis, metalloproteinase production, apoptosis, angiogenesis, proliferation, and differentiation [58,59]. According to Asgari et al., CD147 has a compelling part in cell migration and survival/apoptosis. It can control p53, Bax, and Bcl-2 independently [60].

### 4.3. Role of Angiogenesis Proteins

**Figure 4:**
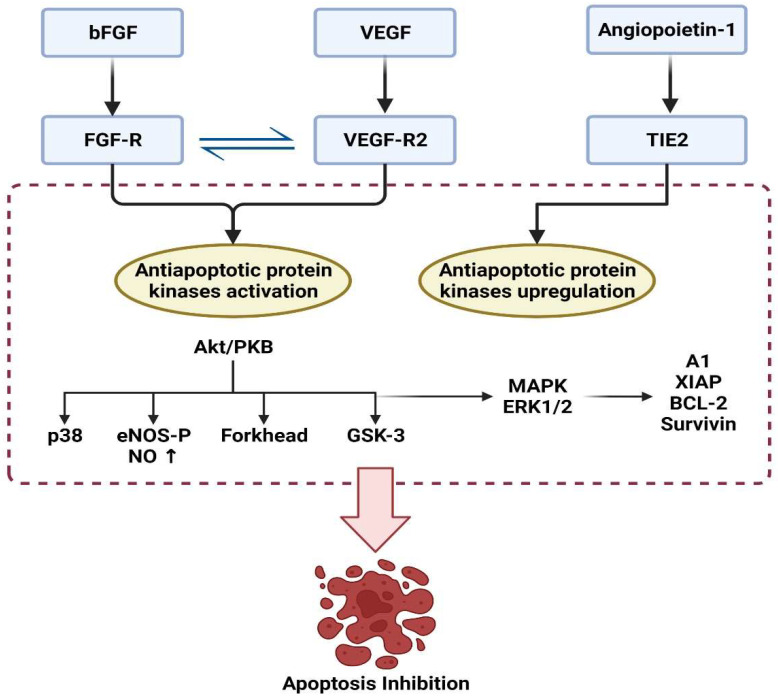
Regulation of apoptosis under the effect of angiogenic factors.

Angiogenesis is the process through which new capillaries develop from pre-existing vessels under different growth factors [61]. Almost all cells survive due to growth factors and interaction with the extracellular matrix. The depletion of growth factors, including VEGF, bFGF, and angiopoietin-1, may cause uncontrolled apoptosis. So, their activation has been shown to promote survival by blocking apoptosis [62]. The action of VEGF is transduced by two tyrosine kinase receptors, flt-1, and flk-1. Cell multiplication and survival have been linked to flk-1, whereas chemotaxis and vascular permeability have been linked to flt-1 [62]. The protein kinase B (or Akt) and MAPK are parts of the flk-1-activated signaling pathway [62,63]. Moreover, VEGF has been demonstrated to enhance cell survival by activating the PI3K/Akt pathway [61,64]. Surprisingly, VEGF’s survival function relies on VEGF binding to VEGFR2 (KDR/flk-1) [64]. Hence, VEGFR2 and PI3K/Akt signal transduction pathways are critical in VEGF-induced survival enhancement. Furthermore, Akt-dependent stimulation of endothelial nitric oxide synthase (NOS) results in increased endothelial NO production, which is one of the downstream effector mechanisms, mediating the antiapoptotic VEGF action [65,66]. Alternatively, the PI3K/Akt pathway promotes survivin transcription and can suppress the p38 mitogen-activated protein kinase (MAPK) [67,68]. The VEGF induces MAPK/ERK pathway stimulation and suppresses the stress activated protein kinase/c-Jun amino-terminal kinase pathway, which is implicated in the antiapoptotic action mediated by VEGF. Interestingly, activation of the PI3K/Akt pathway mediates the survival impact of VEGF on cells and the migrating effect of VEGF via Akt-dependent phosphorylation and eNOS activation [69] (Figure 4).

### 4.4. Role of Apoptotic Proteins

Apoptosis participates in the development, regeneration, and integrity of multicellular organisms. It is critical to multiply, eliminate and maintain homeostatic physiological functions during the transformation, development, and tissue renewal processes [70]. This natural cell death process is genetically predetermined and involves the destruction of cells in response to specific signals under different mediators (Table 1) [71]. However, if this common cell death mechanism fails, the effects might be disastrous. This entire mechanism is interconnected to multiple conserved anticlines. It terminates for stimulating and destroying cellular inputs [72] in different growth plate zones under the effect of pesticides, e.g., thiram. Typically, programmed cellular senescence is regulated by a range of intra and extracellular signals guided by the cell’s surroundings and internal signaling [73]. It has been shown that some proteins hold both pro and anti-apoptotic functions in the cell, which plays a pivotal part in the governance of apoptosis [74]. The apoptosis inducer, mediator, and executioner genes are regularly transduced into those cells to compensate for the absence of the endogenous homologue [75]. Moreover, several factors contribute to the effectiveness of molecular-targeted specific medications; understanding these factors offers insight into an effective treatment plan for designing molecular-targeted medicines [70].

There are two primary apoptotic mechanisms: the intrinsic approach, which involves early mitochondrial disruption caused by cellular stress or cytotoxic assaults, and the extrinsic system, activated by death receptor stimulation [77].

#### 4.4.1. Intrinsic/Mitochondrial-Mediated Proteins

**Figure 5:**
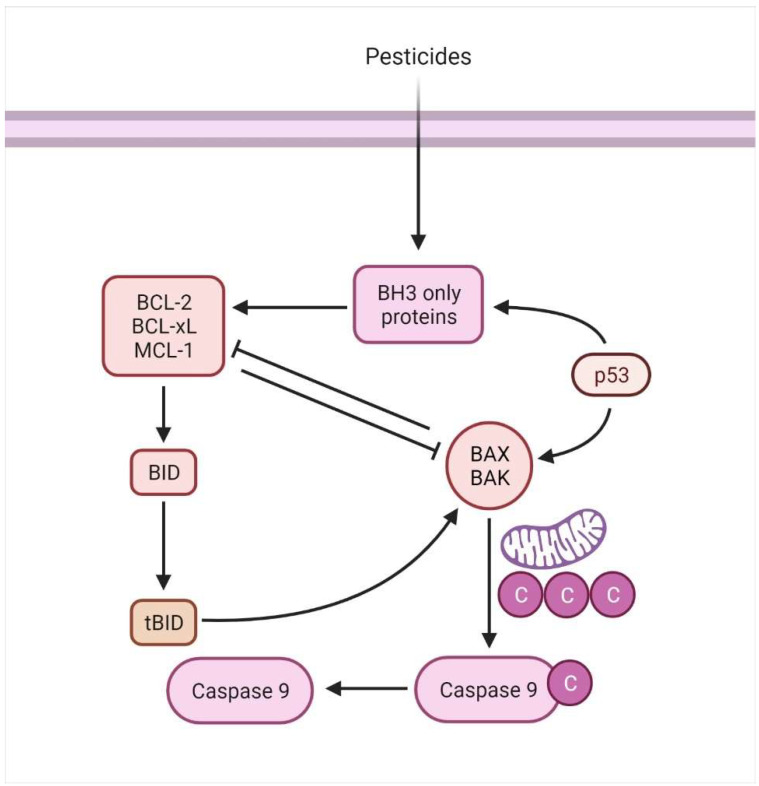
The mitochondrial mediated/intrinsic apoptosis is associated with the activation of BH3 proteins.

The intrinsic mechanism mainly relates to mitochondrial-mediated apoptotic pathways, activated by numerous extra and intracellular stressors, including oxidative damage, irradiation, and cytotoxic medication [78]. Besides being controlled or triggered by extraneous stimulants, apoptosis may also be governed by stimuli such as cellular infliction and oxidative strain [79]. The attributes of the Bcl family (Bax and Bcl-2) are known as pro-apoptotic or anti-apoptotic proteins and are found on the mitochondrial membrane. They are critical mediators of the intrinsic apoptotic process [80], resulting in cytochrome c (Cyto C) release within the cytoplasm upon disrupting the mitochondrial membrane. The clemency of Cyto C within the cytoplasm results in forming a network including APAF1 and pro-caspase 9, which is referred to as an apoptosome (Figure 5). This combination cleaves and activates executioners, such as Caspase-3 and Caspase-7, ensuring cell death at the end of the process [81]. Thus, intrinsic mitochondrial dysfunction leads to decreased inner mitochondrial function, increased superoxide ion synthesis, mitochondrial malfunction, and the affluence of MCG (matrix calcium glutathione) [82].

#### 4.4.2. Bcl-2 Family Proteins

The Bcl-2 proteins family firmly guards against intrinsic or mitochondrial cell death. This family group is unruffled of similar constructional proteins along with antagonistic activities [83]. Initiators, effectors, and anti-apoptotic proteins are all members of the Bcl-2 family. Usually, pro-apoptotic proteins and stimulants counteract apoptotic-promoting activities directly with the anti-apoptotic proteins. When these pro and anti-apoptotic proteins are balanced, a cell’s survival or death is determined [4]. The balance among these proteins is tangled with programmed cell death through the involvement of mitochondria [84]. This is why it’ is essential to keep an eye out for abnormalities that might cause an imbalance in mitochondrial biogenesis, resulting in the destruction of inner membrane potentiality and an upturn in superoxide ions generation [70].

All Bcl-2 proteins have a unique series of homology in sustained areas, called BH (Bcl-2 homology) motifs, that determine shape and activity [85]. All candidates of the anti-apoptotic category and a subgroup of the representative pro-apoptotic type are a multi-domain group of proteins having a series similarity within three to four BH domains. Such a subgroup of pro-apoptotic members termed BH3 exclusive proteins demising domain since mandatory multi-domain Bcl-2 family proteins [86].

#### 4.4.3. Anti-Apoptotic Bcl-2 Proteins

The resistance mechanism against apoptosis is recognized as one of the defining characteristics of cells [87]. Many studies have proved that the increase of anti-apoptotic Bcl-2 proteins plays a central role in B cell lymphomagenesis [88]. Another possibility is that the expression level of these proteins is a sign of how reliant the cell is on the protein to keep itself stable [89]. Apoptosis is inhibited initially by the competence of anti-apoptotic members to pickle and detain the pro-apoptotic proteins (e.g., Bax and Bak), preventing mitochondrial membrane damage [90]. To prevent apoptosis from releasing Cyto C from the membrane, these Bcl-2 family proteins reside on the outer mitochondrial subunit [83].

**Figure 6:**
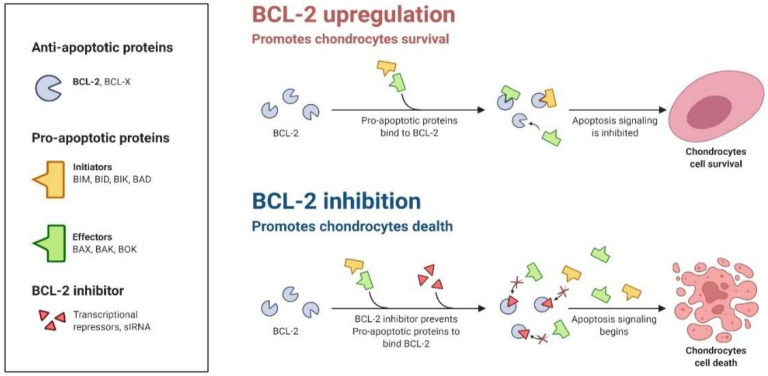
The imbalance or inhibition of anti-apoptotic proteins (e.g., Bcl-2) may initiate apoptosis.

The dysregulation of anti-apoptotic proteins occurs during malignancies that further promote tumor formation [91]. The equilibrium of such proteins is disrupted when Bcl-2 (or related proteins) are dysregulated, or BH3-only proteins or effector proteins are depleted [92] (Figure 6). The numerous genetic pathways behind these anomalies are discussed elsewhere [93]. However, it is critical to recognize that the elevated levels of pro-survival protein members may be an epigenetically mediated adaptive response to cellular stress. In summary, cells with increased Bcl-2 expression function as a safeguard against apoptosis in cellular stressors (Figure 5). As Letai describes, these cells are “primed for death” and should thus be very sensitive to the lack of Bcl-2′s protective role [92].

#### 4.4.4. Pro-Apoptotic Bcl-2 Proteins

The anti-apoptotic proteins contribute to cell continuity by controlling or keeping the levels of pro-apoptotic proteins [94]. In contrast, the pro-apoptotic proteins are members of the Bcl-2 family with several BH domains. These members are structurally related and have pro-survival relatives. During an apoptotic event, Bax and Bak homodimerize and oligomerize in the exterior mitochondrial layer, resulting in the discharge of Cyto C within the cytoplasm [84]. Numerous research has been conducted to determine the prognostic value of pro-apoptotic proteins. For instance, the mRNA intensity of Bax and Bcl-2 was evaluated in colorectal cancer patients. According to the results, Bax substantially induced tumor cell apoptosis and regulated Bcl-2 to prevent apoptosis [95].

The mitochondrial cell death pathway is interceded by multi-domain proteins such as Bax and Bak, with anti-apoptotic proteins. Various genetic and biochemical studies act as upstream regulating entities that resist the intrinsic death-inducing behavior at mitochondrial membranes [96]. Their interaction with mitochondrial membranes has been extensively investigated, revealing that these proteins regulate mitochondrial outer membrane permeability (MOP) for apoptotic proteins’ discharge, e.g., cytochrome [97,98]. Various conditions have been found to activate their action, showing that these pro-apoptotic proteins may remain dormant until triggered. For example, Bax is located in the cytosol instead of interacting membrane organelles before any cell death signal [99]. The presence of Bax in its inactive soluble form shows that the C-terminal membrane-anchoring motif is snuggled into the same niche that most likely engages BH3 peptides [100]. Bax changes its shape in response to still-unknown signals, forming oligomers, revealing its C-terminal membrane-anchoring motif, and entering into mitochondrial membranes [96,101]. Additional proteins (e.g., Bak and Bok) seem to be present in membranes on a constitutive basis. Even if Bax or Bak are embedded in the outer mitochondrial membrane, oligomerization appears to be required to discharge cytochrome c and other apoptotic proteins [96]. Additionally, anti-apoptotic proteins of the Bcl-2 family may create pathological conditions conducive to apoptosis by their capacity to attach and trigger Bax, Bak, and Bok.

#### 4.4.5. Activation of the Executioner Caspase-3 and Caspase-7

**Figure 7:**
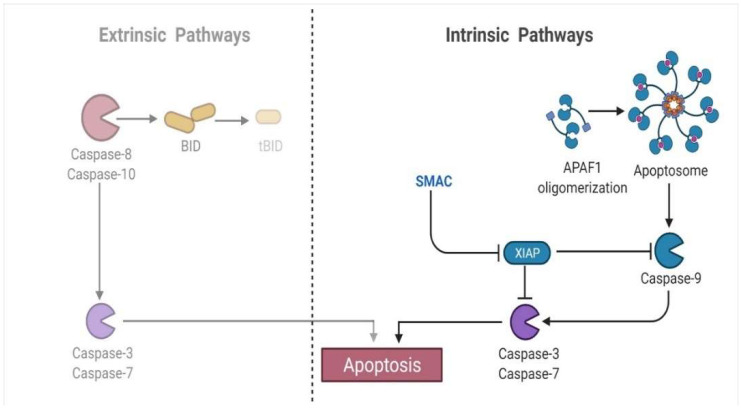
The executioner caspase proteins (e.g., Caspase-3, Caspase-7) trigger apoptosis in both extrinsic and intrinsic pathways.

The execution phase is the last stage in the induction of apoptosis. It is distinguished by vacuolization, chromatin condensation, genomic instability, and blebbing of the cell membrane [82]. It is initiated by a series of events, with the executioner (e.g., caspases-3 and -7) acting as the culmination point of the process [102]. It is thought that the cleavage and activation of executioners cause significant intracellular proteolysis and cellular functioning impairment [103,104]. Additionally, activation and aggregation of these two caspases have already been distinguished as biochemical apoptotic hallmarks [105]. Because of their almost identical activity toward specific synthetic peptide substrates, the primary executioners are widely believed to have functionally similar roles inside the cell death mechanism [106]. As a result, the expression of these executor caspases is critical for providing a reliable biomarker of disease progression. For instance, a study that evaluated Caspase-3 expression in healthy and cancerous prostates concluded that the absence of Caspase-3 in cancerous affected cells may destroy apoptotic components [107] (Figure 7). A study on MCF7 cells discovered that the overexpression of Caspase-3 increases chemosensitivity to develop drug counteraction [108]. Moreover, it is an efficient marker for detecting gastric cancer differentiation, development, invasion, and dissemination through modulation of infiltrating lymphocyte apoptosis [109]. In contrast, Caspase-7 activates the spontaneously anti-parallel complex formation of two precursor variants. Few research studies have investigated the role of this caspase type in apoptosis regulatory oversight and its relationship to clinicopathological characteristics. For example, its reduced protein expression level was found to be a strong predictor in all breast tumors [110]. The findings further indicated that Caspase-7 is abnormally produced and contributes to cellular adhesion and division, making it a potential target for the treatment of different disorders [111].

### 4.5. Role of Inflammasome Proteins

An inflammation is some kind of a defensive immunological response triggered by an innate immune system in feedback to damaging stimuli [112]. Stimulation of the inflammasome complex is triggered when chronic inflammation persists, resulting in the release of pro-inflammatory cytokines (for example, interleukin-1beta) over time. To reduce inflammation and induce cell death, this protein complex has been shown to have a substantial role in carcinogenesis and cancer progression [113]. Hence, it is a crucial modulator of the innate immune system [114].

**Figure 8:**
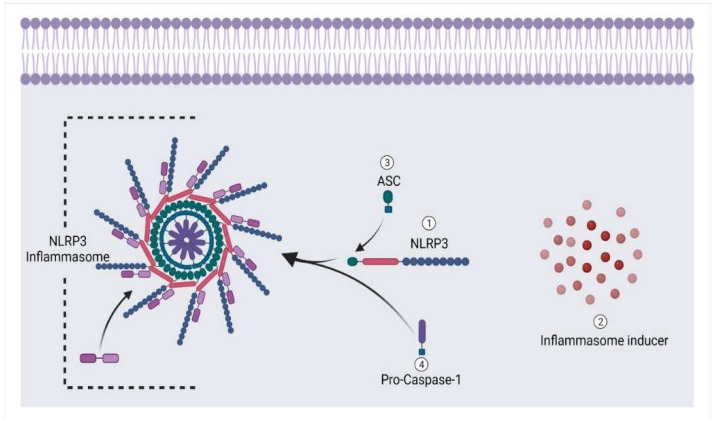
Formation of NLRP3 base inflammasome under the influence of cell damage.

An NLRP3 base inflammasome seems to be a critical part of the innate immunity that various stimuli may trigger. It incorporates NLRP3, an apoptosis-associated speck-like protein with a CARD (ASC), and pro-Caspase-1 [115] (Figure 8). Nevertheless, Caspase-1 is notorious for generating pyroptosis and was first discovered to activate apoptosis under beta cell lymphoma 2 (Bcl-2) inhibition [116]. Moreover, it is involved in apoptosis in several clinical situations [117]. It has recently been discovered that NLRP3 inflammasomes are formed due to intrinsic apoptosis [118,119]. The Caspase-3 and Caspase-7 break down the membrane protein pannexin1 during apoptosis. This helps free pannexin1 channel activity from self-inhibition caused by its cytoplasmic C-terminal tail. After cleaving these executioners, pannexin 1 sends K^+^ out of the cell to activate the NLRP3 inflammasome, which in turn produces IL-1β [120]. So, the NLRP3 base inflammasome has much importance in bone-related disorders. According to research by Kin et al., patients experiencing arthritis had increased expression of the NLRP3 inflammasome, which was associated with elevated pro-inflammatory mediators [121] (Figure 7). Another study by Pan et al. found that nucleosides, including nucleoside analogues, may stimulate host immune responses in mice with type II collagen-induced arthritis by interacting with TREM receptors on the skin and NLRP3 inflammasomes [122]. As a result, combining these pro-inflammatory cytokines may result in phenotypic alterations in cells throughout the ossification process [115,123].

## 5. Prevention and Treatment against Apoptotic Events of TD

According to our previous studies, chlorogenic acid (CGA) in the feed may lower the prevalence of tibial dyschondroplasia as it targets specific mediators related to apoptotic events [12,26,27,45,124]. CGA is the most abundant phenolic acid in nature, being synthesized when quinic and caffeic acids are esterified. It occurs naturally in various fruits, herbs, and vegetables, including kiwi fruit, coffee beans, tobacco leaves, and honeysuckle [125]. It has been seen in pharmacological trials to have significant antioxidant, anti-inflammatory, antiviral, anticancer, cardioprotective, anti-apoptotic, and free radical scavenging properties [27,45,126,127]. Zhang et al. discovered that CGA might stimulate osteoblast growth and speed the S phase transition process. Additionally, it may promote Bcl-2 expression and limit Bax activation during apoptosis, ultimately decreasing osteoblast apoptosis [128]. It has been shown in recent work by Kulyar et al. that CGA has therapeutic benefits for TD chickens by modulating a variety of pathways associated with apoptosis and inflammasome [27,45]. Furthermore, targeting micro RNAs is a better therapy for overcoming such disorders. It is well known that miRNAs control mRNA expression via binding to their 3′-UTRs. These microRNAs (miRNAs) convoluted in various skeletal buildup aspects [129,130,131]. Such miRNAs attach to complemental bases in 3′ untranslated part of particular target mRNAs, preventing the production of specific proteins [132]. The major biological actions such as cell proliferation, apoptosis, cell differentiation, and metabolism are influenced by miRNAs. As a result, miRNA expression alterations may significantly impact normal and abnormal cells [133]. The miR-460a is an essential micro RNA involved in many structural and metabolic cellular processes [134]. Moreover, it is correlated with inflammatory genes, including IL-1β, in broiler chickens [134,135]. Some other options can be used from the treatment perspective (Table 2).

Recent research has focused on the idea that, in contrast to pro-apoptotic, the anti-apoptotic approach in tibial dyschondroplasia may occasionally be advantageous as it reduces the inflammatory response [45]. In fact, an earlier regulation of apoptosis may be beneficial for chondrocytes’ survival. Moreover, local and international industries adopt a proper nutritional strategy for preventing tibial dyschondroplasia (e.g., a proper ratio of calcium, phosphorus, and vitamin D [147,148]) and vaccination for other bone disorders, e.g., viral and bacterial arthritis, chondronecrosis, osteomyelitis, etc. [149,150].

These findings provide fresh knowledge to researchers. Even though several significant research studies have contributed to a deeper understanding of the treatment and prevention of tibial dyschondroplasia, the knowledge is still inadequate for such a critical issue. As a result, the discovery of effective and very sound therapy is urgently required. Moreover, future research on the mechanism of protein-to-protein interaction with the latest scientific findings may lay the foundation for associated bone disorders, e.g., osteoarthritis and osteoporosis.

## 6. Conclusions

Tibial dyschondroplasia (TD) has been the most severe tibiotarsus disease, causing tibial epiphysis in fast-growing chickens. It causes unusual apoptosis in the tibial growth plate (GP), reducing chondrocyte activity and compromising osteogenesis. According to different pathologic findings and molecular mechanisms, apoptosis and inflammasome are critical. Hence, a deep insight into these pathways is necessary to know the most effective therapeutic approach.

## Figures and Tables

**Table 1:** The various mediators of apoptosis and their functions [76].

Mediators			Function
Bcl-2	Intrinsic pathway	Anti-apoptotic mediators	Control permeability of mitochondria
Bcl-xL			Inhibit p53, Bax, Bak, and granzyme B
c-FLIP	Extrinsic pathway		Prevent caspase-8 from binding with different death receptors
NF-κB			Upregulate c-FLIP and IAPs (anti-apoptotic mediators); accelerate growth; activate anti-apoptotic p65 gene regulator
IAP	Alternate pathway		Mimic Bcl-2; inhibit caspase-9
Survivin			Regulate mitosis of cell cycle; inhibit caspases-3 and caspase-7
XIAP			Inhibit caspase-3, caspase-7, caspase-9; activate NF-κB
JAK, STAT	Cytokine receptors		Induction of survival genes through the NF-κB activation
MAPK			Translocate to nucleus; induce anti-apoptotic factors regarding genetic production
PKR			Phosphorylation of protein initiation factor 2 α and IκB kinase complex; delay apoptosis
CDKs, cyclins, and CDK inhibitors			Control machinery of cell cycle
TRAIL	Extrinsic pathway	Pro-apoptotic mediators	A ligand that binds to TNF-α
TNF-α			Bind to TNF-α; breakdown sphingomyelin into ceramide
FasL			Bind to Fas; breakdown sphingomyelin into ceramide
DISC			Activate caspase-8, 10; recruit c-FLIP; cleave tBid for increasing MOMP
TRADD, FADD			Recruit procasapase-8, 10
TWEAK			Ligand that binds to receptors of pro-apoptosis
NGF			Ligand that binds to receptors of pro-apoptosis
BH3-only	Intrinsic mitochondrial pathway		Mediate death stimuli from environment and cell; inactivate Bcl-2, Bcl-xL, and trigger Bax/Bak
Bim			Bind and inhibit Bcl-2 and Bax/Bak
Bmf			Bind and inhibit Bcl-2
Bik, Bad			Bind and inhibit Bcl-2
Bid			Activate tBid, inactive Bcl-2
PUMA, NOXA			Activate Bax for MOMP increase
Bax, Bak			Cause cytochrome c release, caspase-12 activation, and ER depletion of calcium
AIF	Mitochondrial substances		Induce caspase independent condensation of chromatin and DNA fragmentation
Endo G			Break DNA
Smac/DIABLO, HtrA2/Omi			Bind and neutralize IAPs
Procaspases-2,-3,-9			Initiate caspase cascade
Cytochrome c			Reduce mitochondrial-membrane potential; bind to procaspase-9 and Apaf-1 for enhancing apoptosome
ER pathway			TRAF2 dissociation and caspase-12 activation; cytochrome c release
-3, -6, -7	Caspases (effector)		Cleaving proteins of cell membrane, nucleus, and cytoplasm
Granzyme B	Alternate substances		Activate effector of caspases
Ceramide			Inhibit Bcl-2; cytochrome c release; activate caspase-9, activate Bax; release cathepsins
p53			Suppress Bcl-2 transcription; Bax production, insulin growth factor binding protein-3; Fas receptor upregulation
Cathepsin D			Activate procaspase-3, 9; Bid cleavage
c-Abl tyrosine kinase			Cytochrome c release

**Table 2:** Alternative treatment options for controlling apoptotic events in tibial dyschondroplasia.

Name	Active Components	References
*Morinda officinalis*	Iridoids glycoside	[136]
Resveratrol	Phytoalexin, polyphenolic	[137]
Hesperetin	Flavonoids	[138]
Angelica	Ferulic acid, butylidenephthalide, and polysaccharides	[139]
Tetrandrine	Alkaloids	[140]
Puerarin	Isoflavone	[141]
Berberine II	Alkaloids (Isoquinoline)	[142]
Sophoridine	Matrine	[143]
*Bauhinia championii flavone*	Flavonoids	[144]
*Achyranthes bidentata*	Phytosterone, phytoecdysteroids, saccharides and saponins	[145]
Sinomenine	Alkaloids	[146]

## Data Availability

Not applicable.

## Abbreviations

Bcl-2, B-cell lymphoma 2; Bcl-xL, Bcl-2-associated protein xL; Bax, Bcl-2-associated protein x; Bak, Bcl-2-associated protein k; c-FLIP, FLICE-like inhibitory protein; NF-κB, nuclear factor-κB; IκB, inhibitory-κB; IAPs, surviving of apoptosis proteins; XIAP, X-linked inhibitor of apoptosis protein; JAK, Janus kinase; STAT, signal transducers and activators of transcription; MAPK, mitogen-activated protein kinase; PKR, protein kinase R; CDK, cyclin-dependent kinase, TRAIL, tumor necrosis factor-related apoptosis inducing ligand; FasL, Fas ligand; DISC, death-inducing signaling complex; c-FLIP, FLICE-like inhibitory protein; MOMP, mitochondrial outer membrane permeability; FADD, Fas-associated death domain; TRADD, TNFR1-associated death domain; TWEAK, TNF-like WEAK inducer of apoptosis; NGF, nerve growth factor; Bcl-2, B-cell lymphoma 2; BH3, Bcl-homology-3; tBid, truncated Bid; Bax, Bcl-2-associated protein x; Bak, Bcl-2-associated protein k; Apaf-1, apoptotsis activating factor-1—Activates procaspase 9; AIF, apoptosis inducing factor; Endo G, endonuclease G; IAPs, surviving of apoptosis proteins; Smac/DIABLO, second mitochondrial-derived activator of caspases—Director inhibitor of apoptosis-binding protein with Low pI; TRAF2, TNF receptor associated factor 2.
